# Evidence for a Novel Mechanism of Antimicrobial Action of a Cyclic R-,W-Rich Hexapeptide

**DOI:** 10.1371/journal.pone.0125056

**Published:** 2015-04-15

**Authors:** Kathi Scheinpflug, Oxana Krylova, Heike Nikolenko, Charley Thurm, Margitta Dathe

**Affiliations:** Department of Chemical Biology, Leibniz-Institut für Molekulare Pharmakologie (FMP), Berlin, Germany; University of Cambridge, UNITED KINGDOM

## Abstract

The development of antimicrobial peptides as new class of antibiotic agents requires structural characterisation and understanding of their diverse mechanisms of action. As the cyclic hexapeptide cWFW (cyclo(RRRWFW)) does not exert its rapid cell killing activity by membrane permeabilisation, in this study we investigated alternative mechanisms of action, such as peptide translocation into the cytoplasm and peptide interaction with components of the phospholipid matrix of the bacterial membrane. Using fluorescence microscopy and an HPLC-based strategy to analyse peptide uptake into the cells we could confirm the cytoplasmic membrane as the major peptide target. However, unexpectedly we observed accumulation of cWFW at distinct sites of the membrane. Further characterisation of peptide-membrane interaction involved live cell imaging to visualise the distribution of the lipid cardiolipin (CL) and isothermal titration calorimetry to determine the binding affinity to model membranes with different bacterial lipid compositions. Our results demonstrate a distribution of the cyclic peptide similar to that of cardiolipin within the membrane and highly preferred affinity of cWFW for CL-rich phosphatidylethanolamine (POPE) matrices. These observations point to a novel mechanism of antimicrobial killing for the cyclic hexapeptide cWFW which is neither based on membrane permeabilisation nor translocation into the cytoplasm but rather on preferred partitioning into particular lipid domains. As the phospholipids POPE/CL play a key role in the dynamic organisation of bacterial membranes we discuss the consequences of this peptide-lipid-interaction and outline the impact on antimicrobial peptide research.

## Introduction

Considering the increasing number of infections caused by multi-resistant bacteria over the last years, there is a clinical need for the development of antimicrobial peptides (AMPs) as new class of antibiotic agents [1]. As part of the innate immune system those peptides possess a broad-spectrum activity against many different microorganisms thus representing an effective defence system. Their application even exceeds the fight against antibiotic-resistant pathogens as various peptides were also found to modulate host immunity by exerting anti-infective, anti-inflammatory and wound-healing activity [2].

Although high structural diversity has evolved among those molecules, the large number of cationic and hydrophobic residues, as found in the subclass of R-,W-rich peptides, provides the physical prerequisite for interaction with the microbes’ membrane [3, 4]. Electrostatic interactions between positively charged side chains and anionic lipids convey high specificity of AMPs towards bacteria compared to eukaryotic membranes, the latter being composed mainly of zwitterionic lipids. Subsequently, hydrophobic peptide domains are able to protrude into the bilayer and interact with the fatty acid chains. Hence, strong peptide amphipathicity favours the initial contact with bacterial membranes and is considered the basis for the different mechanisms of action that have been suggested for AMPs [5, 6].

The most common way of antimicrobial killing is triggered by disruption of the cytoplasmic membrane, e.g. by pore formation, which is rather unspecific but highly efficient [7, 8]. Alternative mechanisms of action include peptide translocation into the cytoplasm where they interfere with metabolic processes, such as protein synthesis or DNA replication, while some peptides, like the lantibiotic nisin, are known to interact directly with specific membrane components [9–11]. In general, cationic AMPs have been demonstrated to be able to induce anionic lipid clustering which is proposed to trigger bacterial growth arrest or cell death [12, 13]. In this context, disturbance of pre-existing lipid microdomains is discussed to stimulate phase boundary effects that affect membrane integrity. In addition, direct peptide-lipid-interaction might influence overall functionality of protein complexes such as those involved in transport, cell wall synthesis and cell division in bacteria [14–16].

The structure-activity-relationship of small cyclic R-,W-rich peptides is well characterised [3, 4, 17, 18]. Among these, the antimicrobial hexapeptide cWFW (cyclo(RRRWFW)) has been demonstrated to be highly active against Gram positive as well as Gram negative bacteria, while no toxicity towards mammalian cells was observed [19]. With cationic charge and high amphipathicity the cyclic peptide features the structural determinants for membrane activity. However, we could show before that it does not exert its antimicrobial activity by permeabilisation of the cytoplasmic membrane.

In this study we investigated alternative mechanisms of action of cWFW with the aid of specifically designed peptide derivatives [20]. First, confocal laser scanning microscopy (CLSM) was used to visualise peptide translocation into the cytoplasm. An HPLC-based strategy, originally developed to study peptide uptake into eukaryotic cells, was applied to analyse internalised amounts of the cyclic peptide [21]. Further investigation focussed on specific peptide-lipid-interaction as elevated levels of the anionic phospholipid cardiolipin (CL) or its perturbed organisation have been proposed to impair bacterial cell division [22, 23]. Here, we used fluorescence microscopy to reveal peptide effects on the lipid distribution in bacterial membranes and isothermal titration calorimetry (ITC) to determine the binding affinity of cWFW to model membrane systems.

## Materials and Methods

### Peptides, lipids and chemicals

All peptides used in this study were purchased from Biosyntan GmbH (Germany) and prepared by simultaneous multiple peptide synthesis according to SHEPPARD and analysed using mass spectrometry. Peptide purity was determined with >95%. The 3-N-(7-nitrobenz-2-oxa-1,3-diazole-4-yl)-2,3-diaminopropionic acid (Dap-NBD)-bearing analogue, c(RRRW[Dap-NBD]W) was developed for laser scanning microscopy investigations, and the introduction of lysine (K) in c(KRKW[Dap-NBD]W) was required for HPLC-based uptake studies. Both cWFW analogues conserved the non-membrane-permeabilising mode of action of the parent sequence [20]. The N-terminally carboxyfluorescein-labelled Buforin II (Fluos-TRSSRAGLQFPVGRVHRLLRK-NH_2_) served as control.

For liposome preparation we used 1-palmitoyl-2-oleoylphosphatidyl-sn-glycerol (POPG), 1-palmitoyl-2-oleoyl-sn*-*glycero-3-phosphatidylethanolamine (POPE), 1-palmitoyl-2-oleoyl-sn-glycero-3-phosphatidylcholine (POPC), cardiolipin (CL; heart, bovine), *E*. *coli* polar lipid extract (all lipids were purchased from Avanti Polar Lipids, Inc., USA). Consumables used in bacterial cell culture were obtained from TPP (Switzerland). Further chemicals were: BHI (brain-heart-infusion; Becton-Dickinson, Germany), NAO (acridine orange 10-nonyl bromide) and DPBS (Dulbecco’s phosphate buffered saline; both Life Technologies, Germany), phosphate buffer (10 mM NaH_2_PO_4_ /Na_2_HPO_4_, 154 mM NaCl, 0.1 mM Na-EDTA, pH 7.4), agarose, lysogeny broth (LB), o-nitroaniline, penicillin G, yeast extract, sucrose (all from Sigma-Aldrich, Germany), trifluoroacetic acid (TFA, Acros Organics, Belgium), acetonitrile (VWR Chemicals, Germany), Triton X-100 (Merck, Germany), calcein (Fluka, USA).

### Bacterial strains and growth conditions

*E*. *coli* DH5α (E.c.), *B*. *subtilis* DSM 347 and 168 (B.s.) were obtained from DSMZ, Germany. Cell wall-deficient L-form bacteria *E*. *coli* W1655 F+ (LWF+) and *B*. *subtilis* L-170 (L-170) were kindly provided by Dr Christian Hoischen, Leibniz Institute for Age Research, Germany [24].

*E*. *coli* and *B*. *subtilis* were grown to mid-log phase (OD_600_ ± 0.4) in LB medium at 37°C, 180 rpm in 100 ml conical flasks under aerobic conditions (OD_600_ = 1 corresponds to 2.8 × 10^8^ CFU/ml *E*. *coli* and 8.8 × 10^7^ CFU/ml *B*. *subtilis*).

For L-form bacteria BHI medium was supplemented with 70 mg/ml penicillin and additional 50 mg/ml sucrose and 30 mg/ml yeast extract in the case of *B*. *subtilis* L-170. L-form cells were cultivated as described above using flat bottom glass flasks and OD was measured at 550 nm (OD_550_ = 1 corresponds to 3.92 × 10^8^ CFU/ml L-170 and 9.75 × 10^8^ CFU/ml L-WF+).

### Antimicrobial activity

To determine the minimal inhibitory peptide concentration (MIC) of bacterial growth we applied a microdilution technique as described previously [19]. Briefly, the antimicrobial activity was tested against L-form bacteria in 96-well plates with final peptide concentrations ranging from 0.05 to 100 μM. Cultures were diluted to OD_550_ 0.4 in BHI containing strain-specific supplements. Cells were grown for 18 hours at 37°C and 180 rpm. Peptide activity was tested in triplicates in at least three independent experiments.

### Fluorescence microscopy

Cells from an overnight culture were diluted 1:100 in fresh medium and grown to mid-log phase. LB-agarose pads were prepared by adding 1.5% agarose to LB medium and heated until the agarose was completely dissolved. In the case of translocation experiments the solution was slightly cooled down before peptides were added at a final concentration of 12 μM. 100 μl LB-agarose was applied to a cover slip and stored at 8°C in the dark for ~30 minutes. The hardened LB-pad was placed on top of 10 μl of bacterial culture (OD_600_ 0.4) and live cell imaging was performed for up to 100 minutes in a heat controlled chamber to avoid desiccation.

For cardiolipin (CL) staining, NAO was added at a final concentration of 500 nM to 2 ml cell suspension (*B*. *subtilis* 168) adjusted to OD_600_ 0.2 in growth medium. Cells were first incubated with the dye for 60 minutes in the dark, at room temperature at 600 rpm (ensuring sufficient aeration). Afterwards, cWFW was added at a final concentration of 12 μM and co-incubated with NAO for another 30 minutes under the same conditions.

Specific staining of CL with NAO has been shown before and is neither influenced by fixation nor depolarisation of the membrane [25, 26]. The emission shift from green to red fluorescence (513 nm to 640 nm) was reported to be exclusive for binding to CL and is caused by narrow stacking of dye molecules. Images were captured using a Zeiss Axiovert M200 inverted fluorescence microscope and an LSM 510 ConfoCor2 (Germany) with plan-apochromat 63, 100×/1.4 objective, respectively, and processed with ImageJ.

### HPLC uptake studies

As the cytoplasmic membrane of bacteria has been identified as the target of cWFW, cell wall-deficient L-form strains derived from *E*. *coli* (LWF+) and *B*. *subtilis* (L-170) were used to quantify peptide uptake into the cytoplasm [20, 27]. The approach, originally developed for the investigation of peptide uptake into eukaryotic cells, is based on chemical modification of the amino group of lysine side chains with o-nitroaniline and fluorescence-based HPLC detection of non-modified and modified sequences [20, 28]. Fluorescence labelling was necessary for quantification of cyclic hexapeptide uptake.

Cells were cultivated to an OD_550_ ± 0.8 in conical flasks as described above. After harvesting and centrifugation at 2000 × g (Sigma 3K12, Germany) for 12 min and removal of culture medium, cells were washed twice with DPBSG (DPBS containing 1g/l glucose). 10^9^ cells/ml buffer were incubated with 24 nmol (24 μM) Fluos-Buforin II and cR2[NBD], respectively, in Eppendorf tubes for 30 min at 37°C with gentle shaking (Eppendorf Thermomixer comfort, Germany). After centrifugation at 1500 × g for 4 min at 1°C (Biofuge primo R, Heraeus, Germany), the supernatant was removed and cells were washed twice at 1°C with 0.5 ml DPBS. Resuspended cells in 0.5 ml ice-cold DPBS were treated with 10 μl freshly diazotized o-nitroaniline solution for 10 min in an ice bath to modify cell surface-exposed peptide molecules. (Diazotized o-nitroaniline solution was prepared as follows: 35 mg o-nitroaniline were dissolved in 2 ml ethanol before adding 1 ml 0.25 M HCl. 40 mg NaNO_2_ were dissolved in 1 ml H_2_O. The reagent solution was prepared by mixing 400 μl o-nitroaniline solution and 50 μl NaNO_2_ solution for 5 min at room temperature.) After centrifugation and two washing steps with ice-cold DPBS, cell pellets were lysed with 0.5 ml 0.1% Triton X-100 (dissolved in 0.1% trifluoroacetic acid) and stored at -20°C until HPLC analysis. Control samples without o-nitroaniline treatment were prepared under identical conditions and used to determine the total amount of cell-accumulated peptides. Prior to HPLC analysis lysates were thawed to room temperature. Protein content was determined with BCA Protein Assay Kit (Thermo Scientific, Germany) according to manufacturer’s instructions. The average protein content of 10^9^ cells was ~40 μg. Three independent experiments were performed in triplicates.

HPLC analysis was performed as described before using a Jasco LC-2000 Plus (U.S.) with a ProntoSil 300-5-C18-H column (250 × 4.6 mm, 5 μm) (Bischoff Chromatography, Germany) and a precolumn with PolyenCap A300, 10 μm [20]. Peptide amounts were quantified via calibration curves for carboxyfluorescein (ex 445 nm, em 520 nm, gain 1000) and NBD fluorescence (ex 470 nm, em 520 nm, gain 1000), respectively.

### Liposome preparation

Large unilamellar vesicles (LUVs) were used for isothermal titration calorimetry studies and prepared as described before [27]. Briefly, lipids were first dissolved in chloroform and mixed in the desired molar lipid ratios: POPE/CL, POPC/CL (87.5/12.5%) and POPE/POPG (75/25%). As CL potentially carries two negative charges it was applied at half POPG concentrations to give liposomes with identical surface charge [29]. Subsequently, chloroform was evaporated under nitrogen stream and obtained lipid films were dried under high vacuum for several hours and rehydrated in phosphate buffer by thorough vortexing. The solution was heated 2–3 times in a water bath to 40°C to facilitate resuspension. POPE-LUVs were obtained after 35-fold extrusion through two stacked polycarbonate membranes (100 nm pore size) using a mini extruder (Avestin, Switzerland). The procedure gives vesicles with a diameter of 90–120 nm as determined with dynamic light scattering (DLS) performed on a Zetasizer Nano ZS ZEN 3600 (Malvern Instruments, UK). Large liposomes for dye release experiments were prepared with a solution of 80 mM calcein in 10 mM Tris buffer, 0.1 mM EDTA, pH 7.4 and treated to remove non-incorporated dye as described before [27].

The lipid concentration of *E*. *coli* extract preparations was determined from dried lipid films by gravimetric analysis. Liposomes were prepared after resuspension in phosphate buffer and sonication on ice for 25 min under nitrogen stream using a Labsonic L ultrasonicator (B. Braun Biotech, Germany) followed by centrifugation for 5 min at 11000 × g (Eppendorf MiniSpin, Germany) to remove potential titanium debris [30]. The diameter of LUVs from *E*. *coli* extract was 90 nm. According to manufacturer's data, *E*. *coli* polar lipid extract is a three component mixture containing 71.4% PE-, 23.4% PG-lipids and 5.2% CL (mol%).

### Dye release

Peptide-induced calcein release was monitored fluorimetrically by measuring the time-dependent decrease in self-quenching (ex 490 nm, em 514 nm) at room temperature on a LS 50B spectrofluorimeter (Perkin Elmer, Germany) as described before [27]. Lipid concentration was 25 μM in Tris buffer (10 mM Tris, 154 mM NaCl, pH 7.4) and peptide concentrations ranged from 1 to 200 μM. The fluorescence intensity corresponding to 100% release was determined by addition of a 10% Triton X-100 solution. In this study, calcein release was analysed after 1 min of incubation with 10 μM peptide and 25 μM liposomal suspensions.

### Isothermal titration calorimetry

High-sensitivity isothermal titration calorimetry (ITC) experiments to characterise peptide interaction with model membranes were performed at 25°C on a VP-ITC instrument (MicroCall, Malvern Instruments, UK). Binding data were generated as described before [31]. Prior to use, solutions were gently degassed under vacuum. 10 μl aliquots of 5 to 10 mM lipid vesicles were titrated into a 40 μM peptide solution inside the calorimeter cell (1.4 ml) while the reaction mixture was continuously stirred at 307 rpm. Time intervals between injections were adjusted to 10 min which was sufficient for the heat signal to return to baseline level. Automated baseline adjustment and peak integration were accomplished with the public-domain software NITPIC [32]. In control experiments lipid vesicles were injected into buffer without peptide which resulted in small and constant heat of dilution.

Binding parameters, such as hydrophobic partition coefficient (*K*_*0*_) and enthalpy of binding (*ΔH°*), were derived by applying a surface partition equilibrium under the specific condition that peptide adsorption is linearly related to the peptide concentration immediately above the membrane surface. This concentration in close proximity to the membrane is determined by electrostatic interactions and depends on the bulk concentration of peptide, peptide charge and membrane surface potential. Negatively charged lipid layers attract cationic peptides. Consequently, the membrane surface concentration is enhanced compared to that in the bulk solution. After binding of cationic peptides to an electrically neutral membrane it becomes positively charged which results in a repulsion of further peptides from the membrane surface. Using the Gouy—Chapman theory which accounts for electrostatic effects, it is possible to calculate the peptide surface concentration and to determine the partition coefficient *K*_*0*_ [33]. Nonlinear least-squares data fitting was performed with Excel 2010 (Microsoft) using the Solver add-in (Frontline Systems) [34]. The Gibbs free energy was calculated according to *ΔG*° = -R*T* ln(55.5 M × *K*_*0*_), where 55.5 M is the molar concentration of water in the aqueous phase. Finally, the entropic contribution to membrane partitioning,-*TΔS°*, was derived from the Gibbs-Helmholtz equation, *ΔG*° = *ΔH*°-*TΔS*°.

## Results

### Peptide derivatives

The small cyclic hexapeptide cWFW is highly active against Gram positive and Gram negative bacteria. However, we could demonstrate before that the antimicrobial activity is not based on membrane permeabilisation [19]. In order to investigate alternative mechanisms of action, i.e. peptide translocation into the cytoplasm, we used confocal laser scanning microscopy and HPLC. Corresponding analyses required the use of special peptide derivatives which have been characterised before by Scheinpflug et al. [20], (Table 1). For fluorescence microscopy the label NBD was introduced while a lysine containing hexapeptide was required for chemical modification in HPLC-based experiments. cWFW and the two analogues, cW[NBD]W and cR2[NBD], revealed almost identical amphipathicity as demonstrated by HPLC retention times between 17.5 and 17.9 minutes [20]. However, the minimal inhibitory concentration (MIC) of the analogues against *E*. *coli* and *B*. *subtilis* as well as cell wall-deficient L-form bacteria was reduced compared to the parent peptide cWFW. Substitution of the two arginines next to the hydrophobic cluster with lysine led to peptide properties similar to those found for NBD-labelling alone (Table 1), [20]. Although the changes applied to cWFW had an impact on peptide properties, the derivatives, like the parent peptide, did not permeabilise the bacterial membrane at the MIC. Since the integrity of the cytoplasmic membrane appeared to be unaffected the derivatives cW[NBD]W and cR2[NBD] were considered reliable tools for the application in subsequent translocation studies.

**Table 1:** Cyclic hexapeptides used in translocation studies and investigation of phospholipid interaction.

Peptide	Sequence^1^	MIC [μM]				Application
		*E*. *coli*		*B*. *subtilis*		
		DH5α	L-WF+	DSM 347	L-170	
cWFW	c(RRRWFW)	3	12	3	6	parent peptide, ITC
cW[NBD]W	c(RRRW[Dap-NBD]W)	50	50	25	50	CLSM
cR2[NBD]	c(KRKW[Dap-NBD]W)	50	50	12	50	HPLC
F-Buforin II	Fluos-TRSSRAGLQFP VGRVHRLLRK-NH_2_	6	50	6	50	control

Different peptide analogues were synthesised in order to meet the experimental requirements of the methods applied in this study. Determination of the minimal inhibitory concentration (MIC) of the peptides against *E*. *coli* DH5α and *B*. *subtilis* DSM 347 has been reported before [20].

^1^Dap—diaminopropionic acid; Fluos—carboxyfluorescein; NBD—nitrobenzoxadiazole

### Peptide translocation studies with CLSM

**Fig 1:**
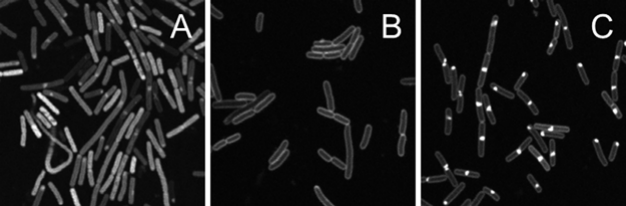
Localisation of antimicrobial peptides in *E*. *coli* and *B*. *subtilis* analysed with fluorescence microscopy. Live cells were incubated with 12 μM peptide for 30 minutes. Localisation studies were performed in at least three independent experiments. Representative images show (A) fluorescein-labelled Buforin II in *B*. *subtilis* which served as control for cytoplasmic peptide localisation, (B) cW[NBD]W in *E*. *coli* and (C) in *B*. *subtilis*. While Buforin II was internalised into the cytoplasm, the cyclic hexapepetide bound to the membrane of Gram positive and Gram negative bacteria. Moreover, the peptide was found to accumulate at septa and polar regions in *B*. *subtilis*.

The localisation of the fluorescent labelled antimicrobial peptide cW[NBD]W was studied in Gram positive and Gram negative bacteria (Fig 1). Buforin II was used as positive control for peptide uptake into the cytoplasm. Its mechanism of action was reported to involve interaction with DNA after translocation over the cytoplasmic membrane without permeabilisation [35, 36]. Fluorescein-labelled Buforin II showed rapid uptake into the cytoplasm of *B*. *subtilis* (Fig 1A). On the contrary, cW[NBD]W fluorescence was not detectable within the cytoplasm of *E*. *coli* and *B*. *subtilis* cells but the peptide accumulated in the bacterial membrane (Fig 1B and 1C). In addition, prominent peptide accumulations at distinct sites were found in Gram positive *B*. *subtilis*. Here, the antimicrobial peptide was enriched in the mid-cell area and the poles, while some cells also showed sub-polar peptide localisation. Similar accumulation of cW[NBD]W was also observed in *E*. *coli* at incubation times longer than 60 min.

### Uptake studies with HPLC

**Fig 2:**
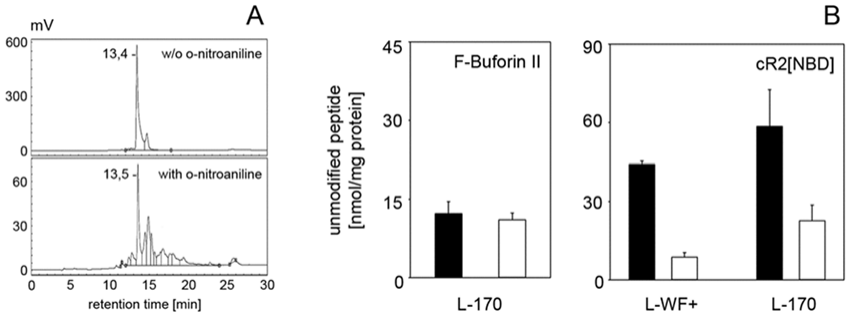
Antimicrobial peptide uptake into cell wall-deficient L-form bacteria. (A) Representative HPLC spectra of cR2[NBD] moieties in *B*. *subtilis* L-170 cells without and after o-nitroaniline treatment. Calibration chromatograms of the peptides dissolved in TFA showed identical retention profiles like peptides in uptake studies without o-nitroaniline exposure. (B) quantification of unmodified peptide: fluorescein-labelled Buforin II (left) and cR2[NBD] (right) after 30 minutes incubation time. Black bars: without o-nitroaniline (total amount of cell-associated peptides), white bars: after o-nitroaniline treatment (amount of peptides not accessible for modification). 10^9^ cells/ml buffer were incubated with 24 nmol (24 μM) Fluos-Buforin II and cR2[NBD], respectively.

Chemical modification with o-nitroaniline was used to distinguish between external and internalised peptides [20]. The highly reactive compound covalently binds to the free amino group in the lysine side chains of cR2[NBD]. Peptide moieties bound to the surface of the membrane are modified while peptides which have penetrated into or across the membrane are not accessible for the o-nitroaniline reaction. HPLC analysis revealed different retention times for modified and unmodified species which allowed quantification of peptide translocation (Fig 2A). In addition to the non-modified peptide several smaller peaks with higher *t*_R_ values were found which result from peptide moieties modified to a different extent. Fig 2B represents the amount of unmodified peptide which was not accessible for the o-nitroaniline reaction. As for fluorescence microscopy, HPLC results with the cyclic peptide were compared to internalisation of Buforin II. Here, we observed similar amounts of unmodified Buforin II in control preparations without o-nitroaniline and after o-nitroaniline treatment. Buforin II was not exposed to the modification reaction which confirms that the control peptide does not accumulate in the membrane but translocates into the cell (Figs 1A and 2B). Concerning cR2[NBD], however, most of the cyclic peptides were accessible for the reaction with o-nitroaniline which is only possible if the peptide is located on the cell surface. The fraction of unmodified cR2[NBD], inaccessible for chemical modification, amounts to about 20% and 30% of the total cell-bound peptides in *E*. *coli* L-WF+ and *B*. *subtilis* L-170, respectively (Fig 2B).

### Peptide influence on lipid distribution

**Fig 3:**
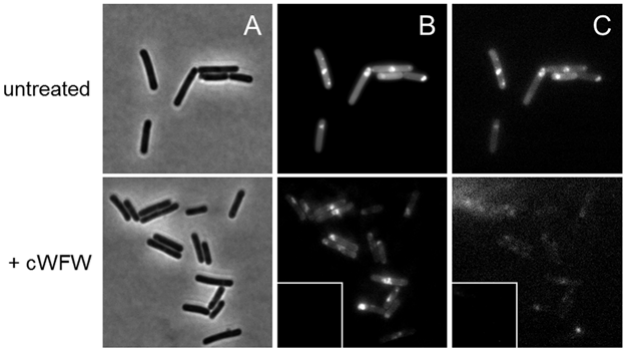
Influence of cWFW on cardiolipin distribution. Cardiolipin staining in *B*. *subtilis* 168 membranes was performed with NAO in untreated control cells and after peptide incubation with 12 μM cWFW. (A) Bright-field, (B) NAO fluorescence at 525 nm (binding to negative phospholipids) and (C) NAO fluorescence at 640 nm (specific interaction with CL). Untreated cells show distinct staining of cardiolipin at the septa and poles. However, a strong decrease in fluorescence intensity was observed in the presence of the antimicrobial peptide cWFW at both wavelengths, insets showing images processed with identical intensity settings as applied for control cells. At 640 nm NAO fluorescence was virtually abolished. Experiments were performed in duplicate, representative images of two independent experiments are shown.

The phospholipid cardiolipin (CL) was shown to be located in the inner and outer leaflet of most bacterial membranes [13]. It accumulates at the poles and division planes and has been suggested to interfere with cell division processes [37, 38]. Due to the particular membrane accumulation of cW[NBD]W in *B*. *subtilis*, we became interested in investigating peptide-lipid-interaction in live cells (Fig 3). In order to visualise CL microdomains we stained the phospholipid with the fluorescent probe NAO [25]. Upon binding to negatively charged phospholipids the dye emits at 520 nm while only specific interaction between NAO and CL is associated with a fluorescence shift from green to red (640 nm) due to tighter stacking of NAO molecules [26]. In untreated cells we observed CL domains at the poles and septa (Fig 3, top row). However, incubation of *B*. *subtilis* with the antimicrobial hexapeptide cWFW resulted in a significant decrease in NAO fluorescence intensity at both wavelengths when analysed using identical intensity settings. Even after appropriate image processing there was hardly any CL staining detectable at 640 nm (Fig 3, bottom row). As the fluorescence signal at 520 nm was also highly reduced NAO binding to other negatively charged lipids was also affected. This observation indicates severe peptide interference with the lipid matrix *in vivo*.

### Peptide interaction with model membranes

The correlation between cW[NBD]W accumulation at septal and polar regions of cells and reported CL localisation led to the hypothesis that this phospholipid might promote peptide adsorption to the bacterial membrane. Dye release experiments performed in addition to earlier studies [27] including also CL-containing liposomes confirmed that cWFW is not able to permeabilise bilayers even at molar peptide/lipid ratios as high as 1/2.5. Permeabilisation followed the order 2% (PE/25%PG)<8% (PE/7.5% CL), (PC/25% PG)<15% (PC/7.5% CL). The lipid systems composed of POPE proved to be resistant towards cWFW action. At a CL content of 12.5% peptide-induced dye release was only 18%. Permeabilisation of POPC-based bilayers decreased with enhanced content of negatively charged POPG [27]. In contrast, at very a high content of cardiolipin (40 mol%) we observed pronounced dye release from POPE- and POPC-based LUVs of more than 70%.

**Fig 4:**
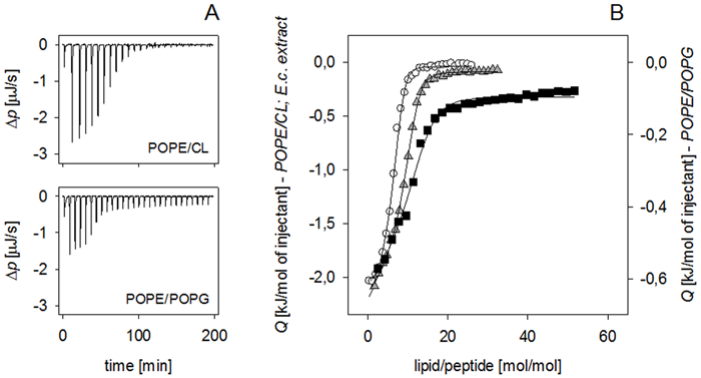
Thermodynamic characterisation of cWFW binding to model membranes of different lipid compositions. LUVs (5 mM POPE/CL, 10 mM POPE/POPG and *E*. *coli* lipid extract) were titrated into 40 μM cWFW solution at 25°C. (A) Representative thermograms of the corresponding ITC titration experiments: differential heating power, *Δp*, versus time, POPE/CL (87.5/12.5 mol%, top) and POPE/POPG (75/25 mol%, bottom). (B) Isotherms of peptide adsorption to negatively charged POPE-based liposomes: integrated and normalised heat of reaction versus lipid/peptide molar ratio, POPE/CL (circles, left ordinate), *E*. *coli* extract (triangles, left ordinate) and POPE/POPG (squares, right ordinate). Solid lines represent best fits to experimental data in terms of a surface partition equilibrium modulated by electrostatic effects [33] assuming a lipid accessibility factor of γ = 0.5 for POPE/POPG, POPC/CL, γ = 0.6 for *E*.*coli* extract and γ = 1 for POPE/CL vesicles (see text for details).

Next, cWFW binding to lipid bilayers was characterised using isothermal titration calorimetry (ITC). We examined the affinity of cWFW to liposomes mimicking the lipid composition of bacterial *E*. *coli* membranes: POPE-based LUVs doped with either 25% POPG or 12.5% CL (net charge -2), both exhibiting the same density of negative charge as well as LUVs from *E*. *coli* lipid extract. POPC-based vesicles containing 12.5% CL were used for comparison. Representative ITC titrations of different lipid vesicles into cWFW solution and the corresponding binding isotherms of peptide adsorption are presented in Fig 4 and Table 2. Perfect fitting of the adsorption data to the binding model assuming a lipid accessibility factor of γ = 0.5 for POPE/POPG and POPC/CL vesicles [33] suggests that only lipids in the outer leaflet are accessible for peptide binding. In the case of POPE/CL vesicles, however, the same assumption did not result in good data fitting. A more accurate fit was obtained with γ = 1 (with fit goodness given as sum of squared deviations: *ΣΔ* ^*2*^ *Q* (J/mol) = 3.84 × 10^–2^ for γ = 1, compared to *ΣΔ* ^*2*^ *Q* (J/mol) = 1.37 × 10^–1^ for γ = 0.5) which points to the negatively charged lipid from both the outer and inner leaflet being involved in cWFW binding.

**Table 2:** Thermodynamic parameters of cWFW binding to LUVs of bacterial membrane-mimicking lipid composition as derived from ITC experiments.

*Lipid composition*	*K*_*0*_ *[M* ^*-1*^ *]*	*ΔG° [kJ/mol]*	*ΔH° [kJ/mol]*	*-TΔS° [kJ/mol]*
POPE/CL (87.5/12.5 mol%)	5.5 × 10^7^	-54.1	-12.0	-42.1
POPE/POPG (75/25 mol%)	3.7 × 10^6^	-47.4	-5.1	-42.3
*E*. *coli* extract	8.8 × 10^6^	-49.6	-16.7	-32.9
POPC/CL (87.5/12.5 mol%)	1.4 × 10^6^	-45.0	-24.3	-20.7

The Gibbs free energy was calculated according to *ΔG*° = -R*T* ln(55.5 M × *K*_*0*_), where 55.5 M is the molar concentration of water in the aqueous phase. The entropic contribution to membrane partitioning,-*TΔS°*, was derived from the Gibbs-Helmholtz equation, *ΔG*° = *ΔH*° - *TΔS*°.

cWFW adsorption to all investigated bilayers was accompanied by an exothermic heat of reaction (Fig 4A and 4B) which is characteristic for membrane partitioning of amphipathic organic molecules and small cyclic peptides [39, 40]. Observed binding constants in the range of 10^6^ M^-1^ reflect strong interactions (Table 2). However, with a *K*_*0*_ value of 5.5 × 10^7^ M^-1^ the affinity of the hexapeptide is more than 10- to 50-fold higher towards CL-containing POPE vesicles compared to POPE/POPG and POPC-based liposomes, respectively. cWFW binding to vesicles of *E*. *coli* lipid extract (*K*_*0*_ = 8.8×10^6^ M^-1^) was slightly less efficient than to CL-doped POPE-vesicles. The high affinity of cWFW to the bacterial model membranes is based on a highly negative Gibbs free energy, *ΔG°*, with both enthalpy and entropy changes favourable for peptide binding (heat release and entropy increase). The entropic and enthalpic contributions observed for peptide binding to the POPC-based bilayer were almost identical (Table 2). However, for all bacterial membrane-mimicking POPE-based lipid systems and *E*. *coli* extract, the entropic impact (*-TΔS°)* on cWFW adsorption was considerably higher than the enthalpic one (*ΔH°)*. Thus, binding of cWFW at POPE systems is an entropy-driven process as expected for molecule partitioning as a result of the classical hydrophobic effect [41]. The hydrophobic effect is determined by the release of water from the hydrophobic side chains of the peptide. Comparing *ΔH°* values, the negative enthalpy change was more pronounced for CL-containing POPE- and POPC-LUVs and vesicles from *E*. *coli* extract (-12.0, -24.3 and -16.7 kJ/mol, respectively) (Table 2). Thus, the strong partitioning of cWFW into the CL-rich POPE bilayer is dominated by a pronounced entropic contribution of peptide binding to POPE systems and a favourable change in enthalpy for CL-containing systems.

## Discussion

The aim of this study was to shed light on the antimicrobial mechanism of action of the cyclic hexapeptide cWFW. With demonstrated high activity against Gram positive as well as Gram negative cells we would have proposed a general killing mechanism, such as disruption of bacterial membranes, which is independent of the presence of outer membrane components or peptidoglycan. However, membrane permeabilisation, as most common mechanism of action of cationic, highly amphipathic AMPs, could not be observed [20]. Here, we investigated peptide translocation into the cytoplasm and interaction with phospholipids of the cytoplasmic membrane which were reported to be crucial steps in alternative modes of antimicrobial action [42, 43].

Using fluorescence microscopy imaging we followed peptide localisation in live bacteria cells. For the membrane-translocating antimicrobial peptide Buforin II we observed rapid uptake into the cytoplasm as reported before [35]. As expected, the control peptide does not accumulate in the cell membrane which was confirmed by our HPLC uptake experiments. This observation is in accordance with binding studies on negative PG/PC and PG/PE model membranes where the overall binding constant of Buforin II with about 2 × 10^5^ M^-1^ is comparably low [44]. In contrast, the major amount of the NBD-labelled cyclic hexapeptide strongly accumulated in the cell membrane. The large differences in the total amount of cell-bound peptide (unmodified cR2[NBD] in experiments without o-nitroaniline) and amount of non-modified peptide detected after treatment with the reagent demonstrate the high preference of the cyclic peptide for the cytoplasmic membrane. Total peptide amounts found to interact with bacterial cells were 4- to 5-fold higher compared to Buforin II. Furthermore, we could not detect any fluorescence of the cyclic peptide within the cytoplasm even at higher concentrations and after more than 60 minutes incubation. Thus, we would propose the non-modified peptides, detected in experiments with o-nitroaniline treatment, to be membrane-bound but not accessible for chemical modification. This implies deep insertion of the hexapeptide into the bilayer. Although the reduced activity found for the newly synthesised peptide derivative might be related to lower initial peptide interaction with the bacterial surface, subsequent steps involved in the mechanism of antimicrobial killing are based on high membrane activity. These results confirm the cytoplasmic membrane as the major target of the R-,W-rich cyclic hexapeptides. Whether translocation of the cyclic peptide to the inner leaflet is involved in the mode of action, as e.g. known for nisin or toroidal pore-forming peptides like magainin, remains to be investigated [11, 45, 46]. Lactoferricin-B peptides, R-,W-rich tritrpticin and indolicidine have been reported to be able to perturb lipid bilayer structures in a way which might be sufficient for spontaneous peptide crossing [47].

Observed cell-specific differences with regard to accumulation and uptake of the cyclic hexapeptide (Figs 1 and 2) are likely to be related to the different lipid composition of the bacterial membranes. The *B*. *subtilis* membrane is composed of about 70 mol% of negatively charged PG and 20 mol% electrically neutral PE, whereas the membrane of *E*. *coli*, with about 25% PG and about 70% PE, contains a significantly higher amount of the neutral negative intrinsic curvature lipid. In addition, both types of bacteria have about 4% negatively charged and negative curvature strain-inducing cardiolipin [13]. The higher content of negatively charged lipids in the membrane of *B*. *subtilis* might drive electrostatic interactions and favour peptide accumulation on the bacterial surface [48]. Preferred peptide binding to membrane regions with high curvature which are rich in cardiolipin microdomains might additionally contribute to the distinct peptide distribution observed in *B*. *subtilis* cells [26, 38, 49].

Staining of cardiolipin domains in the membrane of *B*. *subtilis* with NAO showed typical polar and septal accumulation of the phospholipid (Fig 3, upper row). However, treatment with the antimicrobial hexapeptide cWFW led to a strong decrease of fluorescence intensity. Arouri et al. [50] recently reported on cyclohexapeptide-induced lipid demixing and an enhanced main lipid phase transition temperature of PE/PG bilayers which points to a barrier function-enhancing effect on bacterial membranes. In accordance with these findings, our observation of decreased CL staining after peptide treatment could result from active exclusion of NAO from the membrane due to lipid rearrangement. It remains to be investigated whether cWFW directly prevents the cardiolipin-dye interaction in a competitive manner or peptide-induced lipid demixing leads to changes in dye affinity towards the membrane.

Investigations on the mechanism of action of the cyclic lipopeptide daptomycin showed that the presence of cardiolipin increases binding of the antimicrobial agent to lipid layers but prevents transbilayer movement and pore formation [49]. Our results from titration calorimetry using membrane mimicking lipid systems also suggest a particular role of CL in combination with PE in binding of the cyclic hexapeptide to bacterial membranes. Earlier studies revealed cyclic hexapeptide localisation next to the headgroup/acyl chain interface in PG/PC lipid systems without bilayer permeation even at high molar peptide/lipid ratios [18, 51]. While POPC and POPG form stable bilayers, a central feature of POPE and CL is their tendency to induce negative curvature strain [52, 53]. Our binding studies show that partitioning of cWFW into CL-doped POPE-based bilayers is highly favoured compared to other lipid systems mimicking the bacterial membrane. However, the specificity is not caused by preferred electrostatic attraction to the membrane surface. The pronounced hydrophobic effect reflects a deeper and/or more stable insertion of the peptide into bilayers with a high amount of the negative intrinsic curvature lipid POPE. Additionally, comparing peptide binding to POPE/CL, POPE/POPG bilayers and *E*. *coli* extract, the presence of CL significantly raised the enthalpy released from the system. This increase indicates a higher number of favourable molecular interactions between the peptide and the lipid molecules. Yaroslavov et al. [54] showed that CL can translocate from the inner to outer leaflet of the bilayer in the presence of certain polycationic substances. Considering these observations, cWFW might also be able to induce transbilayer migration of CL to the outer leaflet (CL flip-flop) in POPE/CL membranes. This is consistent with improved data fitting assuming CL flip-flop and underlines the potential contribution of this lipid to peptide binding. When comparing peptide adsorption to lipid vesicles with peptide adsorption to the bacterial membrane, the involved interactions will be driven by the same basic physical principles, though the details may be highly different. Thus, a direct correlation of phenomena observed on model membrane level and the complex biological membrane is often difficult. In the case of our cyclic R-,W-rich hexapeptide, binding studies on liposomes and microscopic studies of live cells point to a particular role of CL in peptide-related reorganisation of the lipid matrix.

We could show that the specificity of the cyclic hexapeptide cWFW is based on pronounced partitioning into lipid layers containing the two negative intrinsic curvature lipids, the electrically neutral POPE and the anionic cardiolipin. The pivotal role of PE and CL in the cell division process has been demonstrated in several studies (for review see Matsumoto *et al*. [16]). It appears that their propensity to form non-bilayer structures is essential for managing the lipid packing constraints at the division site and the cell poles [55]. Besides meeting membrane curvature requirements, both lipids play an important role in the dynamic organisation of bacterial membranes by forming domains which seem to be involved in the recruitment of membrane proteins and the functional regulation of the cell division machinery. For example, the binding affinity of the division plane regulators MinD and MinE to the *E*. *coli* membrane was shown to be dependent on the anionic phospholipids CL and PG [56]. If the antimicrobial hexapeptide cWFW influences CL localisation also *in vivo*, anchoring for those and other peripheral membrane proteins might be disturbed. Furthermore, peptide-induced lipid demixing and enhanced phase rigidity in PE/PG bilayers, as demonstrated by Arouri et al. [50], would interfere with the highly regulated spatial and temporal interplay of cell division proteins. In agreement with such considerations, we observed reduced growth rates of Gram positive and Gram negative bacteria within the first 30 minutes after peptide addition at the MIC followed by complete growth stop.

## Conclusion

We could demonstrate that translocation into the cytoplasm is not a crucial step in the antimicrobial mechanism of action of the cyclic R-,W-rich hexapeptide cWFW.

Our results confirm the bacterial cytoplasmic membrane as target structure and ITC experiments showed high peptide affinity to CL-containing POPE bilayers. We propose that the activity of our cyclic hexapeptides is based on pronounced partitioning into lipid layers containing microdomains of both PE and the anionic cardiolipin. The question raised from this study is how the multiple functions of the lipids, e.g. in cell division, are affected so efficiently that the cells are not able to compensate. Moreover, the molecular details of how phospholipid migration leads to cell death remain to be elucidated. However, favoured peptide interaction with specific lipid domains most likely influences the functional organisation within the bacterial membrane and triggers secondary effects on cell metabolism and homeostasis. Eventually, influencing lipid organisation directly or indirectly by targeting negative intrinsic curvature lipids could be the basis for a highly effective, novel mode of action and a new approach for the design of antimicrobial peptides.
